# The association between exposure to social media alcohol marketing and youth alcohol use behaviors in India and Australia

**DOI:** 10.1186/s12889-018-5645-9

**Published:** 2018-06-13

**Authors:** Himanshu Gupta, Tina Lam, Simone Pettigrew, Robert J. Tait

**Affiliations:** 1 National Drug Research Institute, Faculty of Health Sciences, Curtin University, Perth, WA 6008 Australia; 2 National Drug Research Institute, Faculty of Health Sciences, Curtin University, Perth, WA 6008 Australia

**Keywords:** Alcohol marketing, Internet, Social networking sites, Young people, India, Australia

## Abstract

**Background:**

Alcohol marketing on social networking sites (SNS) is associated with alcohol use among young people. Alcohol companies adapt their online marketing content to specific national contexts and responses to such content differ by national settings. However, there exists very little academic work comparing the association between alcohol marketing on SNS and alcohol use among young people in different national settings and across different SNS. Therefore, we aimed to extend the limited existing work by investigating and comparing the association between self-reported exposure to alcohol marketing on three leading SNS (Facebook, YouTube, and Twitter) and alcohol use among young people in diverse national contexts (India and Australia).

**Methods:**

Cross-sectional, self-report data were obtained from a convenience sample of 631 respondents (330 in India; 301 in Australia) aged 13–25 years via online surveys. Respondents answered questions on their drinking behaviors and involvement with alcohol marketing on SNS.

**Results:**

Many respondents from both countries reported interacting with alcohol content online, predominantly on Facebook, followed by YouTube and then Twitter. The interaction was primarily in the forms of posting/liking/sharing/commenting on items posted on alcohol companies’ social media accounts, viewing the event page/attending the event advertised by an alcohol company via social media, and/or accessing an alcohol website. Multivariate analyses demonstrated significant associations between respondents’ interaction with alcohol content and drinking levels, with effects differing by SNS, demographic group, and country. For example, having friends who shared alcohol-related content was an important predictor of usual alcohol consumption for Indian respondents (*p* < .001), whereas posting alcohol-related information themselves was a stronger predictor among Australians (*p* < .001).

**Conclusions:**

The results suggest that interaction with alcohol-related content on SNS is associated with young people’s alcohol use behaviors and that these behaviors vary by national settings. This study extends previous work by demonstrating this connection across varying social media platforms and national contexts. The results highlight the need to formulate and implement strategies to effectively regulate the SNS alcohol marketing, especially among younger SNS users.

## Background

Alcohol is one of the leading risk factors for global disease burden among the 15–49 years age group [1]. Alcohol use by young people, especially those under 20 years of age, increases the likelihood of risky behaviors such as aggressive incidents and unprotected sex, and adverse outcomes such as learning difficulties, depression, and accidents [2–4].

Accessing social networking sites (SNS) has become a common pastime for young people, with these websites considered an integral part of their leisure and friendship networks [5, 6]. This development has provided alcohol companies with an opportunity to utilize SNS as highly effective platforms to reach this group and promote their products [7].

Alcohol companies post content on their official SNS pages, which previous research suggests is deemed pleasurable and socially desirable by SNS users [8, 9]. This process involves initiating conversations between SNS users and brands, and thus facilitates creation of user-generated content. Prompted by brands, users participate in conversations and post content relating to their real-world activities and socio-cultural identities [6]. For example, posting pictures (sometimes with alcohol), tagging their SNS friends in such posts, and checking-in at events on SNS (e.g., music, fashion, sports, and cultural events created by alcohol companies). This is beneficial to brands because such events are deemed socially desirable, increase social capital, and make younger SNS users less critical of these marketing techniques. This process further facilitates the flow of this content into online users’ peer networks and the generation of more content that is favorable to brands [10, 11].

The studies examining the effects of exposure to alcohol marketing on SNS indicate that exposed youth are likely to develop positive attitudes towards alcohol use [5, 12], regularly consume alcohol [13], engage in heavy and risky drinking [14], and experience subsequent alcohol-related problems/disorders [15].

### Purpose of the study

India and Australia were chosen for this study based on the contrasting alcohol consumption features – 1) dry (India) versus wet (Australia) drinking cultures, 2) proportion of drinkers and/or per capita consumption increasing (India) versus decreasing (Australia) over the past decade, and 3) substantially different socio-cultural norms towards alcohol consumption. These features are discussed at length in the next section.

Harms related to underage drinking are a major problem in Australia [16] and a growing concern in India [1], and are likely influenced by exposure to alcohol marketing [1]. Most of the research exploring the association between alcohol marketing on SNS and alcohol use among young people has been conducted in the USA [12, 15], the UK [6, 14], and Australia [10, 17–21]. However, these studies explored these associations primarily on Facebook, with work involving other SNS such as Twitter and YouTube is sparse. Further, work in other national contexts such as India appears to be lacking, with a few studies identified [22, 23]. Jones et al. (2016) found significant and positive associations between reported exposure to alcohol advertising and branding on Facebook and reported drinking frequency and volume among 16–24 year old Facebook users [19]. Similarly, another study reported that exposure to Internet advertising was significantly associated with frequency of alcohol consumption among 12–17 year old Australians, with these associations varying across age and gender sub-groups [20].

As stated earlier, alcohol companies use marketing strategies on SNS that are tailored to specific national contexts and users’ responses to such marketing content differ by national settings [22–24]. These studies identified common strategies used for alcohol promotion across social media in both countries. These included prompts to engage in time- and event-specific drinking (e.g., “it’s Friday, and it’s beer-o’clock”), alcohol sponsorship of sporting, music, and fashion events, cocktail recipes and food-drink combinations, competitions, brand-related giveaways, and the use of memes. Other strategies were largely country specific, such as posting content relating to inspirational talks, livelihood skills, and sexually suggestive content on Indian social media sites versus references to a brand’s tradition or heritage on Australian sites. Notably, some of the identified strategies (e.g., brand-sponsored events and posts relating to competitions and giveaways) were traditionally more popular amongst younger people. User engagement was assessed through responses to content posted by brands, for example, user-generated messages, images, and videos posted on brands’ social media pages.

However, cross-national comparisons examining the effects of exposure to alcohol marketing on SNS and alcohol use among young people and across different SNS are scant. To extend this work, the present study investigated associations between 13 and 25 year olds’ exposure to and interaction with SNS-based alcohol marketing and alcohol use (assessed as usual consumption) in diverse national contexts (India and Australia), and across varying SNS. The selected SNS were all popular amongst young people, allowed for a variety of comparisons with existing work from the USA [12, 15], the UK [6, 14], and Australia [10, 17–21] and represented SNS not yet examined within the Australian and Indian literature. This information is important to guide the development of regulatory frameworks to minimize any harmful use of alcohol among young people that may result from exposure to alcohol marketing.

### Socio-cultural norms and drinking prevalence and patterns

India and Australia have substantially dissimilar socio-cultural contexts and histories, and represent widely contrasting drinking cultures. In India, 11% of males and 1% of females aged 15–19 years and 29% of males and 2% of females aged 20–24 years report consuming alcohol in the past 12 months [25]. In contrast, in Australia 23% of those aged 12–17 years and 62% of those aged 18–24 years consumed alcohol in the past 12 months, with consumption rates being similar for males and females [26]. Similarly, heavy drinking is less prevalent in India than Australia. For example, about 4% of 18–24 year old Indians are classed as ‘heavy drinkers’, defined as consuming at least 50 g of pure alcohol in a single session at least once a month [27]. By comparison, in the same age group, 42% of Australians reported consuming 5 or more standard drinks (equivalent of 50 + g of alcohol) on a single drinking occasion at least once a month [26].

These differences in national consumption rates are likely due to a range of factors. Socio-cultural norms in India are less accepting of alcohol [28], especially in the context of female drinking [29], and religious practices that proscribe alcohol use (14% of the Indian population is Muslim) [28, 30]. The legal drinking age in India ranges from 18 to 25 years, with sales banned to the whole population in certain states [31]. However, with urbanization and industrialization, economic transition, exposure through extensive alcohol marketing, increased availability, and relaxation of overseas trade rules; alcohol use is increasing in India [31, 32]. Hence, both traditions and social change influence drinking culture in modern India [28, 33, 34]. In contrast, although alcohol has been a ubiquitous feature of Australian culture from its colonial era onwards, per capita consumption has been decreasing over the past 10 years [26, 35]. Consequently, although drinking is more prevalent in Australia compared to India, the drinking trajectory is on the rise in India and on decline in Australia [25, 26].

### SNS use and regulatory environment

Reflecting the size of the overall populations, the sheer number of social media users varies greatly between India and Australia. Twitter has an estimated 23 million users in India (2% of the population) and 3 million in Australia (13% of the population), YouTube has about 40 million users in India (3% of the population) and 14 million in Australia (58% of the population), while Facebook has about 108 million users in India (9% of the population) and 11 million in Australia (46% of the population) [36–38]. Although different SNS vary in terms of user demographics, SNS use overall is more prevalent in the younger age groups. For example, in India about 46% of Facebook users are estimated to be 18–24 years of age and a further 11% are 17 years or younger [39], while 70% of YouTube users are below 35 years of age [40]. Similarly, 36% of Australian Facebook users belong to the 13–24 years age group [38] and about 51% of Australians aged 14–17 years reported using YouTube in 2013 [41]. Among Australian SNS users, Twitter is used by 25% males and 14% females. About 33% of them belong to the 18–29 years age group [42]. Although demographic data on Indian Twitter users are not publically available, it is suggested that about 80% of Twitter users are male [43].

The Advertising Standard Council of India (ASCI) regulates alcohol advertising in India. Although it bans both direct and indirect alcohol advertising in traditional media, it does not cover online alcohol advertising [44]. Therefore, alcohol advertising on SNS remains unfettered and is thus extensive in India [31]. In Australia, alcohol advertising (including on digital media) is self-regulated by the alcohol industry via the Alcohol Beverages Advertising Code [45]. However, the current code only relates to the content of alcohol advertisements, does not consider issues around placement of advertisements on digital media (including where there are no age restrictions), and has very weak enforcement powers [46]. Hence, in both countries SNS are largely unregulated platforms for alcohol companies to promote their products.

## Methods

### Respondents

Eligible respondents were those aged 13–25 years who had lived in India or Australia in the past 12 months, had Internet access, and could understand written English. The choice of this age range was guided by the World Health Organization’s and United Nations’ definition of adolescence and youth [47].

Two online surveys were developed, one for each country, that included items relating to demographic characteristics, drinking patterns (defined below), and involvement with alcohol marketing on Facebook, YouTube, and Twitter. There did not appear any academic study and/or national surveys that sought information relating to the objectives of the present survey. Hence, relevant items from existing studies and national surveys were reviewed and included in the present surveys. To cover the topics identified in the objectives, additional items were developed and included in the surveys. Questions on country-specific types of alcohol differed between the surveys because the types of alcoholic beverages consumed differ between countries. A standard drink chart was provided to inform respondents of standard drink measures (one standard drink = 10 g of alcohol in both countries) to facilitate accurate self-reporting of alcohol consumption levels. The surveys were pilot tested with 20 eligible respondents from each country (10 each in the 13–17 and 18–25 years age groups). The surveys were delivered using Qualtrics Survey Software between March and October 2016.

Survey advertisements were posted on social networking sites, sports clubs’ official social media pages, and youth organizations’ official social media pages, to recruit the respondents. Sports clubs and youth organizations also sent out study invitations via email to their members. Alcohol- and drug-related websites and fora (e.g., Family drug support, Bluelight, and Pill Reports) and a word-of-mouth strategy were also utilized to increase recruitment. Ethics approval was obtained from a University Human Research Ethics Committee and all respondents provided online informed consent. Qualtrics survey software’s ‘anti ballot-stuffing’ option was used to prevent the same IP address from accessing the survey more than once, to prevent duplicate survey entries. Further, as no incentive was offered to the respondents, it was unlikely that an individual respondent would complete the survey more than once, thus further decreasing the chances for duplicate survey entries. Providing an incentive for survey participation would also require collecting respondents’ potentially identifiable information, such as an email address, which could compromise their anonymity. As alcohol consumption is a relatively sensitive issue in India, especially among young people, we chose to keep the participants’ information non-identifiable.

Based on a power calculation, 385 respondents were needed for the survey, from each country (95% CI, with a 5% margin of error and response distribution of 50%) [48]. In total, 680 people opened the survey link of whom, 631 (330 Indians and 301 Australians) respondents who met the eligibility criteria were included in the analysis. The survey response rate was 93% (631/680).

### Measures and statistical analyses

Initially, descriptive analyses were conducted to explore overall patterns in the survey data. The key demographic variables (e.g., age, sex, and education) plus alcohol variables are reported in Table 1, and the key independent variables (e.g., SNS engagement) are reported in Table 2. The outcome measure was the number of usual drinks consumed on a typical drinking day. Usual alcohol consumption was analyzed as the arithmetic mean of the range of the survey responses levels (1/2, 1, 2, 3–4, 5–6, 7–8, 9–10, 11–12, 13–15, and 16–19 drinks) other than 20 or more drinks (coded as 20.5 drinks, winsorizing the data). Non-drinkers’ data were coded as 0. This item was taken from Australia’s largest and longest running alcohol and other drug survey with participants aged 12 years and older [49].

**Table 1:** Respondent demographics

Variable	India n (%)					Australia n (%)				
	Males 13–17 years	Males 18–25 years	Females 13–17 years	Females 18–25 years	Total	Males 13–17 years	Males 18–25 years	Females 13–17 years	Females 18–25 years	Total
Number of respondents	70 (21)	141 (43)	29 (9)	90 (27)	330 (100)	45 (15)	70 (23)	29 (10)	157 (52)	301 (100)
Citizenship of host country ^‡^	70 (100)	140 (99)	29 (100)	90 (100)	329 (100)	40 (89)	53 (76)	25 (86)	127 (81)	245 (82)
Education ^‡^										
Primary (up to yr. 5)	1 (1)	–	1 (3)	–	2 (1)	9 (20)	–	5 (17)	–	14 (5)
Secondary (yr 6–10)	43 (61)	–	18 (62)	–	61 (20)	29 (64)	1 (1)	23 (79)	2 (1)	55 (18)
Secondary (yr 11–12)	26 (27)	34 (25)	10 (35)	11 (12)	81 (25)	7 (16)	42 (60)	1 (23)	71 (45)	121 (40)
University	–	106 (75)	–	79 (88)	185 (56)	–	27 (39)	–	84 (54)	110 (37)
Home Internet connection (yes)	67 (96) ^a^	133 (95)	24 (83) ^a,^*	87 (97) *	311 (94)^!^	45 (100)	70 (100)	28 (97)	153 (98)	296 (98)^!^
Internet hours/day Median (IQR)	1.5 *** (0.5–2.0)	3.5 *** (1.5–3.5)	1.5 ** (1.5–1.5)	3.5 ** (1.5–3.5)	1.5^†††^ (1.5–3.5)	3.5 * (3.5–5.0)	6.5 ^b,^ * (3.5–6.5)	3.5 (3.5–6.5)	3.5 ^b^ (3.5–6.5)	3.5 ^†††^ (3.5–6.5)
Lifetime consumption of alcohol (yes)	33 (47) ***	115 (82) ***	15 (52) *	70 (78) *	233 (71)^!!!^	37 (82) *	67 (96) *	20 (69) **	145 (92) **	269 (89)^!!!^
Any alcohol in the last 4 weeks (yes)	15 (21) *	79 (56) *	6 (21)	42 (47)	142 (43)^!!!^	30 (67) ^b^	58 (83)	9 (31)^b,^***	123 (78)***	220 (86)^!!!^
Usual drinks Median (IQR)	3.5 *** (2.0–4.5)	5.5 ^b,^ *** (3.5–7.5)	3.5 (1.75–5.5)	3.0 ^b^ (2.0–5.5)	4.5^†††^ (3.5–7.5)	3.5 (2.0–4.0)	3.5 ^c^ (2.0–7.5)	1.0 *** (0.0–2.0)	2.0 ^c,^ *** (1.0–3.5)	2.75 ^†††^ (1.0–3.5)

^‡^ Age-group and gender differences not tested; IQR = inter-quartile range; usual drinks = standard (10 g) drinks; yr. = year

Between country comparison (medians) ^†††^ Mann-Whitney *U p* ≤ .001: (frequencies)^!^ Fisher’s exact *p* ≤ .05^!!!^ Fisher’s exact *p* ≤ .001:

(Within country and gender) Between age group comparison: * Mann-Whitney U *p* < .05; ** Mann-Whitney *U p* ≤ .01; *** Mann-Whitney *U p* ≤ .001

(Within country) Between gender comparisons: ^a^ Mann-Whitney U *p* < .05; ^b^ Mann-Whitney *U p* < .01; ^c^ Mann-Whitney *U p* < .001

**Table 2:** Descriptive data on variables included in the multivariate analyses

	India n (%)					Australia n (%)				
	Males 13–17 years	Males 18–25 years	Females 13–17 years	Females18–25 years	Total	Males 13–17 years	Males 18–25 years	Females13–17 years	Females 18–25 years	Total
Trends (increased) in alcohol advertising										
Facebook	40 (57)	93 (66)	15 (52)	54 (60)	202 (63) ^†††^	28 (62) ***	24 (34) ***	12 (41) **	39 (25) **	103 (37) ^†††^
YouTube	34 (49) *	96 (68) *	15 (52)	58 (64)	203 (66) ^†††^	28 (62) **	28 (40) **^, b^	12 (41) *	28 (18) *^, b^	96 (35) ^†††^
Twitter	16 (23) ***	70 (50) ***	5 (17) **	35 (39) **	126 (42) ^†††^	11 (24) *	9 (13) *	4 (14)	8 (5)	32 (12) ^†††^
Alcohol brand logos on merchandise (very often or often)	67 (96)	130 (92)	26 (90)	74 (82)	297 (92) ^†††^	27 (60)	35 (50)	15 (52)	85 (54)	162 (57) ^†††^
Alcohol events attended (Yes)	27 (39)	46 (37)	11 (38)	29 (32)	113 (36) ^†††^	20 (44)	35 (50)	8 (27) *	80 (51) *	143 (51) ^†††^
Suggestions on SNS (less than once a week or weekly)										
Facebook	49 (70)	106 (75) ^b^	18 (62)	49 (54) ^b^	222 (69) ^†††^	25 (55)	31 (44)	14 (48)	67 (43)	137 (49) ^†††^
YouTube	33 (45) **	90 (71) **	17 (59)	52 (58)	202 (66) ^†††^	23 (51)	25 (36)	12 (41)	47 (30)	107 (39) ^†††^
Twitter	25 (36) ***	88 (62) ***	15 (52)	41 (45)	169 (55) ^†††^	8 (18)	10 (14)	6 (20)	17 (11)	41 (15) ^†††^
Sharing own alcohol-related content on SNS (very often or often)										
Facebook	46 (66)	90 (64) ^a^	18 (62)	44 (49) ^a^	198 (65) ^†††^	3 (7)	7 (10)	2 (7)	17 (11)	29 (11) ^†††^
YouTube	33 (47)	87 (62)	15 (52)	44 (49)	179 (65) ^†††^	1 (2)	4 (6)	1 (3)	6 (4)	12 (8) ^†††^
Twitter	38 (54)	84 (60)	16 (55)	45 (50)	183 (82) ^†††^	0 (0)	3 (4)	0 (0)	1 (1)	4 (6) ^†††^
Friends sharing alcohol-related content on SNS (very often or often)										
Facebook	55 (78)	97 (69)	19 (66)	53 (59)	224 (72) ^†††^	15 (33)	31 (44)	7 (24)	81 (51)	134 (51) ^†††^
YouTube	38 (53)	87 (62)	15 (52)	39 (43)	179 (60) ^†††^	12 (27)	9 (13)	6 (21)	11 (7)	15 (38) ^†††^
Twitter	36 (51)	81 (57)	15 (52)	42 (46)	174 (58) ^†††^	1 (2)	4 (6)	0 (0)	9 (6)	14 (5) ^†††^
Noticing alcohol-related content on SNS (very often or often										
Facebook	56 (80)	114 (74)	22 (76)	59 (66)	241 (75) ^†††^	13 (29)	26 (37)	8 (28)	64 (41)	111 (40) ^†††^
YouTube	43 (61)	91 (65)	19 (66)	56 (62)	209 (68) ^†††^	12 (27	29 (42)	8 (62)	44 (28)	93 (33) ^†††^
Twitter	31 (34) *	86 (61) *	15 (52)	49 (54)	181 (59) ^†††^	0 (0)	5 (7)	2 (7)	6 (4)	13 (6) ^†††^

Between country comparison^†††^ Fisher’s exact *p* ≤ .001:

Fisher’s exact (2 sided) between age group (within country and gender) significant difference: * *p* < 0.05; ** < 0.01; *** < 0.001

Fisher’s exact (2 sided) between gender (within country and age group) significant difference: ^a^ *p* < 0.05; ^b^ < 0.01; ^c^ < 0.001

The results are presented in Table 1 stratified by country, gender, and age group (13–17 years, 18–25 years). Age was split at the boundary of the legal purchase age of alcohol (18 years) in Australia (and some Indian states). In addition, marked differences in patterns of alcohol use were anticipated across the age range. Differences in usual drinks consumed were assessed using Mann-Whitney U tests due to the skewed data distributions. Results were compared between age-groups and between genders within each country and finally between countries. Descriptive data on SNS interactions (Table 2) and other differences in proportions were evaluated with Fisher’s exact test due to the low frequencies in some cells.

To examine the relationship between respondents’ alcohol use and involvement with alcohol marketing on SNS, we used a standard model building approach (Hosmer and Lemeshow, 2000). Adjusting for key demographic factors (age, gender, and education), each variable was entered separately with the outcome variable (usual consumption levels). Those with a *p* value of <.20 were retained for multivariate analysis [19]. This process was conducted separately for the Indian and Australian data.

Six multiple linear regression analyses were conducted to assess the associations for the outcome variable for each country for each of the three SNS. Therefore, to adjust for multiple significance testing we only interpret values of *p* ≤ .008 (0.05/6). For each analysis, multivariate outliers were identified and excluded using Mahalanobis distance (*p* < .001); excluded cases ranged from 0 to 3 per analysis. Due to the high correlation between age and education level, the multivariate analyses were repeated excluding education. No variable moved across the threshold (*p* ≤ .008) for interpretation in the second analyses compared with the first. Only the first set of analyses is reported.

The following variables were included in the multivariate analyses. The daily hours spent on the Internet were analyzed as the mean of the range of the survey response levels: < 1 (coded as 0.5), 1–2, 3–4, 5–8, and > 8 (coded as 8.5 h thus winsorizing the data). Perceived trends in alcohol advertising and other alcohol-related marketing efforts on SNS in the last 12 months were coded into four categories (increased, unchanged/no difference, decreased, and don’t use this channel). Frequency of noticing alcohol brand logos on merchandise shown on SNS was dichotomized (due to small cell sizes when analyzed by age-group and sex) as ‘very often or often’ and ‘sometimes, rarely, never’. Information on whether respondents had attended events in the last 12 months (social, music, sports, and other) sponsored by alcohol companies via advertising on SNS (sometimes termed ‘real-world tie-ins’) was analyzed as a dichotomized variable again due to small cell sizes when stratified (attended any event - yes/no).

The frequency of receiving suggestions on each of the three SNS to like or to follow alcohol company pages or to participate in alcohol-related events was coded as ‘less than once a week’, ‘weekly’, ‘every fortnight’, ‘monthly’, and ‘don’t use this channel’. Information on the degree to which respondents posted and viewed alcohol-related content on each SNS was dichotomized (due to small cell sizes) as ‘very often or often’ and ‘sometimes, rarely, or never’. Finally, the degree to which the respondents’ friends or contacts post alcohol-related content on each SNS was coded in the same fashion. SPSS version 22 software was utilized for data analysis. Missing values were managed by using a listwise-deletion approach.

## Results

### Demographics and alcohol consumption

In the Indian sample (*n* = 330), 36% were female and the median age was 20 years (IQR 17–22 years). In the Australian sample, 62% were female and the median age was 20 years (IQR 18–23). Most respondents who completed the Australian survey identified themselves as Australian citizens (82%), whereas nearly all (99%) who completed the Indian version were Indian nationals.

The majority of the Indian (93%) and nearly all Australian respondents (99%) reported having an Internet connection at home. The median daily Internet use was estimated at 1.5 and 3.5 h among younger and older Indians (across gender), respectively. In Australia, usage was 3.5 h across demographic groups, except for older males where it was estimated at 6.5 h (Table 1). Overall, the median time on the Internet was significantly lower in India than Australia (median 1.5 vs 3.5 h *U* 6.8, *p* < .001).

The mean age of first full serve of alcohol was 16.5 (SD 3.5) years in India and 15.2 (SD 2.8) years in Australia, with the earliest age being eight years in both countries. The lifetime prevalence of alcohol use was lower among younger Indians (47% for males and 52% for females) compared to their Australian equivalents (82% for males and 69% for females). Similar patterns were observed for recent drinking (i.e., in the last 4 weeks), across demographic groups and countries (Table 1). The overall prevalence for lifetime and recent drinking was significantly lower in India than Australia (Fisher’s Exact test *p* < .001 for both measures).

The median usual drinking levels were significantly higher among males than females in both countries (India median 5.5 vs 3.5: Australia median 3.5 vs 2.0; Table 1). Further, older Indian males drank significantly more than their younger counterparts (5.5 vs 3.5 drinks) and older Australian females had significantly higher usual drinking (2.0 vs 1.0 drinks) than their younger equivalents. Overall alcohol consumption quantity was significantly higher in India than Australia (median 4.5 vs 2.75 drinks *U* 10.6 *p* < .001). A post-hoc analysis controlling for Internet use, age, and gender found that Indian usual alcohol consumption was still significantly higher than Australian usual consumption (*F* = 13.0 (1508) *p* < .001).

### Exposure to and interaction with alcohol marketing on SNS

Descriptive data on the variables related to exposure to alcohol marketing on SNS and included in the multivariate analyses are presented in Table 2. Across all these variables, Indian respondents reported significantly greater involvement with alcohol-related marketing. Perceived trends in alcohol advertising differed by country. In Australia, the younger participants reported seeing significantly increased amounts of alcohol advertising and other alcohol-related marketing efforts on SNS in the last 12 months. In contrast, older Indians noticed a larger increase than younger Indians.

In both India and Australia, older respondents reported receiving more frequent suggestions on SNS to like or to follow pages or to participate in events with alcohol-related content compared to younger respondents. Finally, on most variables there was greater sharing or interaction on Facebook, followed by YouTube and then Twitter, and this was consistent across demographic groups and countries.

### Associations between usual alcohol consumption and alcohol-related interactions on SNS

Multivariate analyses were conducted to determine which influences were significantly associated with usual alcohol consumption quantity. In total, six multivariate models were developed (two countries by three SNS platforms, as items were specific to each SNS).

In addition to age, gender, and education, the Indian univariate analyses identified the following eight variables for inclusion in the multivariate analyses: (i) daily hours spent on the Internet; (ii) noticing alcohol brand logos on merchandise shown on SNS; (iii) attending alcohol industry sponsored events, (iv) sharing own alcohol-related information on SNS; (v) receiving suggestions to interact with alcohol-related content; (vi) perceived changing trends in alcohol advertising; (vii) respondents’ SNS friends/contacts sharing alcohol-related information on the particular SNS; and (viii) noticing alcohol-related information on SNS.

All three Indian multivariate models were significant, and accounted for 23–28% of variation in usual alcohol use. Only one variable, age, was associated with usual alcohol quantity across the three analyses, controlling for all other factors (*p* < .004-*p* < .003). In the Facebook and Twitter models, consumption increased by about 1/3rd of a drink for each year increase in age, but in the YouTube model it decreased by the same amount for each year of age.

There were three other significant associations in Indian multivariate analyses. First, alcohol use decreased by about 1.3 drinks for each decrease in the frequency measure of friends sharing alcohol-related information on Facebook and YouTube (*p* < .001) (e.g., a decrease from ‘sometimes’ to ‘rarely’). Second, sharing personal alcohol-related content on Twitter (*p* = .003) was associated within an increase in alcohol use of 3.1 drinks for each increase in the frequency of sharing information (e.g., from ‘never/ rarely/ sometimes’ to ‘often/very often’) (Table 3).

**Table 3:** Multivariate associations between select characteristics and usual consumption levels (number of drinks consumed on a typical day) for Indian participants ^a^

Variable	Facebook *n* = 217 B (s.e.) *p*	YouTube *n* = 198 B (s.e.) *p*	Twitter *n* = 168 B (s.e.) *p*
Model Statistics	F(11, 205) 8.896 *p* < .001, AR^2^ .287	F(11, 185) 6.370 *p* < .001, AR^2^ .232	F(11, 156) 5.434 *p* < .001, AR^2^ .226
Model variables			
Age	.315 (.103) .003*	−.326 (.110) .004*	.367(.122) .003*
Gender	−.851 (.405) .037	−.757 (.444) .090	−1.091 (.494) .029
Education	.174 (.436) .691	.247 (.488) .613	−.086 (.531) .871
Internet hours/day	.129 (.117) .271	−.080 (.128) .533	−.307 (.164) .824
Alcohol brand logos on merchandise	.033 (1.227) .979	1.723 (1.326) .195	−.308(3.106) .921
Attended alcohol events advertised on SNS	.778 (.407) .057	1.163 (.455) .011	.876 (.498) .080
Sharing own alcohol-related content on SNS	1.201 (.680) .079	1.453 (.854) .803	3.138 (1.028) .003*
Suggestions on SNS to like/follow alcohol-related content	−.126 (.229) .581	.522 (.258) .044	.191 (.270) .480
Perceived increasing trends in alcohol advertising	.313 (.371) .399	.696 (.528) .189	.064 (.430) .882
Friends sharing alcohol-related information on SNS	−1.343 (.325) < .001*	− 1.451 (.306) < .001*	−.622 (.397) .118
Noticing alcohol-related content on SNS	.221 (.278) .428	.753 (.322) .775	.098 (.341) .020

*AR*^*2*^ adjusted r square, *B* unstandardized coefficient, *s.e.* standard error

^a^ non-drinkers coded as 0 drinks

*p ≤ .008

Each of the overall models for the Australian analyses was significant, accounting for between 13 and 40% of the variance in alcohol use. In addition to age, gender, and education, univariate analyses identified seven variables for inclusion in the multivariate analyses: (i) noticing alcohol brand logos on merchandise shown on SNS; (ii) attending alcohol industry sponsored events, (iii) sharing own alcohol-related information on SNS; (iv) receiving suggestions to interact with alcohol-related content; (v) perceived changing trends in alcohol advertising; (vi) respondents’ SNS friends/contacts sharing alcohol-related information on the particular SNS; and (vii) noticing alcohol-related information on SNS.

In the Australian multivariate analyses, sharing alcohol-related content on Facebook and Twitter (*p* < .001) increased the number of standard drinks consumed by 2.6 and 4.5 drinks respectively for each increase in the frequency of sharing information (e.g., from ‘never/rarely/sometimes’ to ‘often/very often’). Significant associations were also found between gender and usual alcohol consumption across SNS (all *p* < .001) (Table 4), with females drinking between 1.7 and 2.9 fewer drinks than males.

**Table 4:** Multivariate association between select characteristics and usual consumption levels (number of drinks consumed on a typical day) for Australian participants ^a^

	Facebook *n* = 246 B (s.e.) *p*	YouTube *n* = 151 B (s.e.) *p*	Twitter *n* = 65 B (s.e.) *p*
Model Statistics	F(9, 237) 7.205 *p* < .001, AR^2^ .185	F(9, 143) 3.597 *p* < .001, AR^2^ .133	F(7, 63) 7.731 *p* < .001, AR^2^ .402
Model variables			
Age	−.096 (.097) .328	−.200 (.137) .146	−.128 (.180) .478
Gender	−1.76 (.387) <.001*	−1.821 (.478) < .001*	−2.957 (.672) < .001*
Education	.769 (.374) .041	1.220 (.512) .019	1.173 (.677) .092
Alcohol brand logos on merchandise	−.308 (.376) .919	.312 (.486) .522	.439 (.686) .824
Attended alcohol events advertised on SNS	.482 (.370) .194	.110 (.466) .814	−.237 (.677) .728
Sharing own alcohol-related content on SNS	2.448 (.573) < .001*	1.453 (.854) .091	4.484 (1.320) .001*
Suggestions on SNS to like/follow alcohol-related content	−.297 (.147) .044	−.356 (.206) .086	–
Perceived increasing trends in alcohol advertising	–	−.133 (.388) .732	−.808 (.437) .069
Friends sharing alcohol-related information on SNS	−.263 (.207) .205	−.231 (.265) .384	–
Noticing alcohol-related content on SNS	−.032 (.192) .869	–	–

*AR*^*2*^ adjusted r square, *B* unstandardized coefficient, *s.e.* standard error

^a^ non-drinkers coded as 0 drinks

*p ≤ .008

## Discussion

To our knowledge, this is the first cross-national comparison exploring associations between involvement with SNS-based alcohol marketing on three different SNS and young people’s alcohol use, in different national settings.

As per existing data [1], the Indian cohort was expected to have lower consumption than the Australian sample. However, we found higher usual consumption (but lower prevalence) in the Indian cohort than the Australian sample. This discrepancy is likely due to the small sample used in this study, the reliance on those who could read/write English, the non-representative form of sampling, and a potentially more affluent cohort (about 48% of Indian Internet users belong to medium and high status groups) [50] than the general population. Consistent with existing research [25], we found statistically significant gender differences in the quantity consumed, with Indian females drinking less than males, but not in the expected difference in prevalence of drinking. This is potentially attributed to rapidly changing attitudes towards female alcohol use in India, especially among higher status groups [32]. Hence, these findings warrant more research exploring alcohol use among higher status groups and those with high levels of education, in India. In contrast, Australian females reported significantly lower prevalence and quantity drunk compared to males, which is consistent with the results of national surveys [26].

Previous studies have reported significant associations between interaction with alcohol marketing on SNS and more frequent alcohol use and intentions to drink [13, 19, 51]. Consistent with these studies, consumption levels were found to be significantly associated with (i) respondents sharing their own alcohol-related information on SNS and (ii) respondents’ SNS friends/contacts sharing such information on SNS. In India, having friends who shared alcohol-related content was an important predictor of usual drinking, whereas posting alcohol-related information themselves was a stronger predictor of usual drinking among Australian respondents. These national differences likely relate to substantially lower levels of social acceptance of alcohol use in India compared with Australia, hence Indian respondents reported sharing less of their own alcohol-related content on SNS. The above mentioned studies demonstrated these associations only on Facebook, thus the results of this study extend previous work by also demonstrating significant associations between interaction with alcohol-related content on YouTube and Twitter, with different effects found by media type, demographic group, and country.

Overall, the evidence from both countries indicates that many young people are likely to be exposed to alcohol-related marketing on SNS, illustrating the need for comprehensive mandatory regulation. In India, there are currently no regulations on social media alcohol marketing, giving alcohol companies unfettered opportunity to expose youth to alcohol promotion via SNS. This highlights the need to introduce effective strategies to regulate online alcohol marketing to minimize exposure. In the Australian context, high levels of youth involvement with SNS-based alcohol marketing can be at least partially attributed to the deficiencies in the existing code that regulates marketing on social media in Australia [46]. Although a revised version of the code with somewhat stronger placement guidelines is being introduced in Australia in November 2017 [52], it still does not effectively address online alcohol advertising. This indicates the need to further strengthen this code, particularly in relation to addressing exposure of younger SNS users to alcohol marketing online.

Some limitations should be considered while interpreting the results of this study. The samples may not be representative of the Indian and Australian populations in the given age groups. In particular, the respondents had to have Internet access and to be proficient in English. This is likely to be a limitation for the Indian sample, as we do not have information on English language fluency among Indians in the specified age group. However, English is the second official language of India, with about 125 million (out of a billion people) English speaking Indians in 2001 across all ages [53]. Nonetheless, English is the main language for the Internet in India. The relatively small number of Australian users of Twitter limits the conclusions that can be drawn about that SNS. Further, gender differences in response rates are likely related to typically higher female survey participation in Australia and higher male drinking prevalence in India. Although we used a convenience sampling approach, the findings were largely consistent with the body of literature regarding country-level consumption behaviors and the associations between alcohol advertising and youth drinking. As noted in the methods section, this study was slightly underpowered, hence a larger sample would be useful to assess the reliability of our exploratory work, in the future studies. Finally, as this study utilized a cross-sectional study design, it was not possible to determine any causal relationships between exposure to alcohol marketing on SNS and alcohol use. This indicates the need for longitudinal studies that can establish the temporal ordering between these two behaviors. As the results differed between countries, indicating that different mechanisms are likely at play, this study also highlights the need to conduct further cross-national comparisons to explore how social media marketing of alcohol affects alcohol consumption in diverse geographic contexts.

## Conclusions

This is the first study to investigate SNS-based alcohol marketing separately on Facebook, YouTube, and Twitter, among young people in different national contexts. Significant associations identified between alcohol marketing on SNS and youth drinking highlight the need to introduce effective regulations pertaining to alcohol marketing on social media platforms to minimize exposure among younger SNS users.

## Abbreviations

ABAC: Alcohol Beverages Advertising Code
ABS: Australian Bureau of Statistics
AIHW: Australian Institute of Health and Welfare
ASCI: Advertising Standard Council of India
IIPS: International Institute for Population Sciences
SNS: Social Networking Sites
WHO: World Health Organization
